# Transcriptome analysis of mRNA and microRNAs in intramuscular fat tissues of castrated and intact male Chinese Qinchuan cattle

**DOI:** 10.1371/journal.pone.0185961

**Published:** 2017-10-26

**Authors:** Ying-Ying Zhang, Hong-Bao Wang, Ya-Ning Wang, Hong-Cheng Wang, Song Zhang, Jie-Yun Hong, Hong-Fang Guo, Dai Chen, Yang Yang, Lin-Sen Zan

**Affiliations:** 1 College of Animal Science and Technology, Northwest A&F University, Yangling, Shaanxi, China; 2 National Beef Cattle Improvement Center of Northwest A&F University, Yangling, Shaanxi, China; 3 Animal Husbandry and Veterinary Research Institute, Shanghai Academy of Agricultural Sciences, Shanghai, China; 4 NovelBio Bio-Pharm Technology Co., Ltd., Shanghai, China; University of Illinois, UNITED STATES

## Abstract

Intramuscular fat (IMF) is known to enhance beef palatability and can be markedly increased by castration. However, there is little understanding of the molecular mechanism underlying the IMF deposition after castration of beef cattle. We hypothesize that genetic regulators function differently in IMF from bulls and steers. Therefore, after detecting serum testosterone and lipid parameter, as well as the contents of IMF at 6, 12, 18 and 24 months, we have investigated differentially expressed (DE) microRNAs (miRNAs) and mRNAs in IMF of bulls and steers at 24 months of age in Qinchuan cattle using next-generation sequencing, and then explored the possible biopathways regulating IMF deposition. Serum testosterone levels were significantly decreased in steers, whereas IMF content, serum total cholesterol (TC), low-density lipoprotein cholesterol (LDL-C) and triglycerides (TGs) were markedly increased in steers. Comparing the results of steers and bulls, 580 upregulated genes and 1,120 downregulated genes in IMF tissues were identified as DE genes correlated with IMF deposition. The upregulated genes were mainly associated with lipid metabolism, lipogenesis and fatty acid transportation signalling pathways, and the downregulated genes were correlated with immune response and intracellular signal transduction. Concurrently, the DE miRNAs—important players in adipose tissue accumulation induced by castration—were also examined in IMF tissues; 52 DE miRNAs were identified. The expression profiles of selected genes and miRNAs were also confirmed by quantitative real-time PCR (qRT-PCR) assays. Using integrated analysis, we constructed the microRNA-target regulatory network which was supported by target validation using the dual luciferase reporter system. Moreover, Ingenuity Pathway Analysis (IPA) software was used to construct a molecular interaction network that could be involved in regulating IMF after castration. The detected molecular network is closely associated with lipid metabolism and adipocyte differentiation, which is supported by functional identification results of *bta-let-7i* on bovine preadipocytes. These results provided valuable insights into the molecular mechanisms of the IMF phenotype differences between steers and bulls.

## Background

The amount of intramuscular fat (IMF) in a cross-section of muscle tissue is highly correlated with meat palatability, affecting the juiciness, tenderness and flavor. Beef with excellent marbling characteristic could increase the profits of beef producers [1,2]. In the beef industry, efforts to improve production efficiency and select the animals with high-lean growth rate have negatively influenced the deposition of IMF [1,2]. Enhancing intramuscular adipose deposition while maintaining stable levels of lipid deposition in other depots have become an important issue in beef industry. Therefore, it is essential to advance our understanding of the molecular mechanisms that affect IMF deposition so that genomic selection can be used to produce high-grade beef with consistent quality. Regardless of breed, age and diet, gender can contribute to remarkable variation in their IMF content [3,4]. Steers and bulls are good animal models for the comparative study of IMF differences. However, the mechanisms underlying differences in the deposition of IMF after castration are poorly understood.

Cai *et al*. [5] have found that castration aggravates hypercholesterolemia and hepatic steatosis in pigs fed a high-cholesterol diet; these effects can be reversed by testosterone replacement therapy. They identified 19,199 DE genes, although most were related to hepatic lipid metabolism and immune responses. In cattle, Bong *et al*. [6] showed that castration upregulated the lipogenic gene expression of *acetyl-CoA carboxylase* (*ACC*) and *fatty acid synthase* (*FASN*), whereas it downregulated the lipolytic gene expression of *adipose triglyceride lipase* (*ATGL*) and *monoglyceride lipase* (*MGL*) in the *longissimus dorsi* muscle (LM) of castrated Korean cattle. These results suggest that castration contributes to improved marbling through increased lipid uptake and lipogenesis, and decreased lipolysis [6]. Moreover, a comparison of transcriptomes through a customized bovine CombiMatrix oligonucleotide microarray analysis indicated that castration alters several pathways, including adipogenesis, fatty acid oxidation, tricarboxylic acid cycle, and oxidative phosphorylation pathways in the LM of Korean cattle, with *perilipin*-*2* (*PLIN2*), *visfatin*, *1-acylglycerol-3-phosphate-O-acyltransferase* (*AGPAT5*), and many fatty acid oxidation-related genes being upregulated in steers [7]. Furthermore, 1,416 DE genes identified in the subcutaneous fat of Qinchuan cattle between bulls and steers using next-generation sequencing were mainly found to be involved in the steroid hormone stimulus response and lipid response processes [8]. However, accurately identifying major candidate genes that affect IMF through transcriptome analysis of muscle or subcutaneous fat is difficult because of the heterogeneity of skeletal muscle composition and significant transcriptomic differences between subcutaneous fat and IMF [1, 9].

MicroRNAs (miRNAs) are small RNA molecules of 18–23 nucleotides in length that have a vital regulatory role in a post-transcriptional mechanism [10]; an association of miRNAs with meat quality and fat deposits induced by castration has been reported [11–13]. In cattle, the miRCURY^TM^ LNA array was employed to identify 13 DE miRNAs genes between stressed and control Angus cattle with different beef quality [11], implying the vital role of miRNAs on meat quality and beef tenderness. In pigs, 18 miRNAs identified by microarray analysis of the subcutaneous adipose tissue of intact male and castrated male pigs with different backfat thicknesses were primarily involved in the regulation of fatty acid metabolism [12]. Moreover, a high-throughput supported oligo-ligation detection sequencing approach was used to identify 366 unique miRNA genes between castrated and intact full-sib pairs of male pigs with different backfat thicknesses. These genes were involved in proliferation, apoptosis, differentiation, migration and adipose tissue development, suggesting an important role of miRNAs in fat deposition after castration [13]. However, to our knowledge, the relationship between bovine IMF and miRNA expression in steers has not previously been examined.

We hypothesize that testosterone deficiency induced by castration may directly regulated transcription of certain miRNAs, which results in a different IMF deposition phenotype. We therefore compared the expression of miRNAs and mRNAs between steers and bulls to identify IMF-deposition-related miRNAs after castration, and found potential mechanisms contributing to the IMF deposition differences between them. The purpose was to gain further insight into IMF deposition-related miRNAs in cattle, which should improve our understanding of IMF deposition after castration.

## Materials and methods

### Ethics statement

The cattle were born, raised and maintained at the National Beef Cattle Improvement Centre (Yangling, China) and slaughtered using mechanized slaughter line at Shaanxi Qinbao Animal Husbandry Development Co., Ltd. The muscle and fat samples were got from slaughter house. Blood samples were collected according to the guidelines established by the regulations for Administration of Affairs Concerning Experimental Animals (Ministry of Science and Technology, China, 2004) and approved by the Institutional Animal Care and Use Committee (College of Animal and Science and Technology, Northwest A&F University, China). The bull’s castration was performed under local anesthetic. All animals were humanely treated to ameliorate suffering.

### Sample collection

Chinese Qinchuan cattle (n = 12) born within a 30-day period were randomly chosen and divided into 2 groups. The first group comprised 6 males and the second group other 6 males that had been surgically castrated under local anesthetic at 6 months. The cattle with similar genetic backgrounds, produced by one sire, were born, raised and maintained at the National Beef Cattle Improvement Centre (Yangling, China). The genetic resemblance among individuals permits us to better control the cause of variation between experimental clusters and individuals. All animals received the same diet until terminating. The animals were weaned at 3 months of age and fed a diet with a 3:7 concentrate-to-forage ratio until 6 months of age. Then, the 2 groups were fed concentrates consisting of 14% crude protein (CP) and 70% total digestible nutrients (TDN) until they reached 12 months of age, followed by 12% CP and 71% TDN until 18 months, and finally 10% CP and 72% TDN until 24 months. Fasting blood samples were collected prior to castration and semi-annually throughout the study. At 24 months, the animals were stunned with a captive bolt and slaughtered when they weighed 626.1 ± 26.4 kg (bull group) and 531.5 ± 19.8 kg (steer group) at Shaanxi Qinbao Animal Husbandry Development Co., Ltd. Due to the difficulty to separate IMF tissues from LM in Qinchuan cattle, the *sternomandibularis* muscle tissue of each animal was sampled as described by Duarte *et al*., and the IMF tissue was quickly dissected with sufficient care to avoid contaminating the muscle samples with other tissues [14]. All tissue samples were promptly frozen in liquid nitrogen and stored at -80°C for further analysis.

### IMF content, serum testosterone levels and serum lipid measurements

The IMF content was analyzed as previously described [15], and serum was separated from the blood samples also as previously described [5]. Testosterone was measured at 6, 12, 18, and 24 months using a commercial RIA kit (Tianjin Jiuding Medicine Biological Technology Co., Ltd. Tianjin, China). ApoAI and apoB were measured immunoturbidimetrically using commercial reagent kits (Shanghai Fosun Long March Medical Science Co., Ltd. Shanghai, China). High-density lipoprotein cholesterol (HDL-C) and low-density lipoprotein cholesterol (LDL-C) were measured by a direct method using a commercially available kit (Zhejiang Erkn Biological Technology Co., Ltd. Wenzhou, China). Total cholesterol (TC) and triglycerides (TGs) were analyzed using commercial enzymatic (COD-PAP) kits (Shanghai Kehua Biological Engineering Co., Ltd. Shanghai, China).

### Construction of mRNA and miRNA libraries and sequencing

Each sample was ground to powder in liquid nitrogen in a mortar and then was centrifuged in RNase-free tubes treated with TRIzol^®^ Reagent (Invitrogen, Carlsbad, CA, USA). Total RNA extraction from samples was performed according to the manufacturer’s standard instructions (Invitrogen) and then the RNA was prepared and purified using the NucleoSpin^®^ RNA clean up kit (MACHEREY-NAGEL, Germany).RNA concentration was assessed by an Agilent 2200 system (Agilent, USA). RNA quality was determined by formaldehyde denaturation electrophoresis and only those samples showing no degradation (ratios approaching 2:1 for the 28S and 18S bands) were used. All samples had a RNA integrity number (RIN) value of >7. To reduce variation among individuals within each of the 2 groups, total RNA from cattle of the same group was pooled in equal amounts to generate a mixed sample for mRNA library constructions [16,17]. Sequencing libraries were prepared using the Ion Total RNA-Seq Kit v2.0 (Life Technologies, Carlsbad, CA, USA). cDNA libraries were processed for the Proton sequencing process according to the commercially available protocols. Briefly, the cDNA samples were diluted to 2–3 nM with RNase-free water. Then the diluted samples were mixed, the mixture was processed on a OneTouch 2 instrument and enriched on a OneTouch 2 ES station for preparing the temple-positive Ion PI Ion Sphere Particles according to Ion PI Template OT2 200 Kit v2.0 (all from Life Technologies, Carlsbad, CA, USA). The enriched and mixed template-positive Ion PI Ion Sphere Particles of samples was loaded on to 1 P 1v2 Proton and sequenced on Proton Sequencers according to Ion PI Sequencing 200 Kit v2.0 (Life Technologies, Carlsbad, CA, USA).

Four miRNA libraries were constructed. After extracting total RNAs from IMF tissues of all 6 steers and 6 bulls, low molecular weight RNAs were separated on 15% polyacrylamide gels by electrophoresis and miRNA was purified with a miRNeasy Mini Kit (Qiagen, Valencia, CA, USA). A total of 4 small RNA libraries (n = 3 for each libraries) were constructed using an Illumina^®^ TruSeq^TM^ Small RNA Sample Preparation kit (Illumina, San Diego, CA) and sequenced on the HiSeq 2500 Platform (NovelBio Corp. Laboratory, Shanghai). RNA and miRNA sequencing data have been deposited in the National Center for Biotechnology Information (NCBI) Gene Expression Omnibus database (GEO: GSE75063).

### Data analysis of mRNA expression

The sequencing quality was estimated using FastQC software (http://www.bioinformatics.bbsrc.ac.uk/projects/download.html#). Before read mapping, the Clean reads were retrieved from the Raw reads by removing the adaptor sequences, reads with >5% ambiguous bases (noted as N) and low-quality containing >20% bases with a quality of <13. The clean mRNA reads were aligned to the bovine genome (version: Bos 4.6.1) using the MapSplice program (v2.1.8) [18]. The EBseq algorithm was used to filter the DE genes between the steers and bulls groups based on the significant analysis and false discovery rate (FDR) analysis under the following criteria: (1) fold change (FC) >2 or < -2; (2) FDR <0.05 [19,20].

### Analysis of Gene category (GO), and the Kyoto Encyclopedia of Genes and Genomes (KEGG) pathway

Gene category analysis was used to elucidate the biological implications of DE genes according to the NCBI (http://www.ncbi.nlm.nih.gov/), UniProt (http://www.uniprot.org/), and GO (http://www.geneontology.org/), which provides key functional classifications for genes [21]. Similarly, the significant pathways of the DE genes were computed with the KEGG database (http://www.genome.jp/kegg) by using pathway analysis [22,23]. Fisher’s exact test was usually used to filter the GO category and the significant pathways, and FDR was applied to correct the P-values [24,25]. Enrichment analysis was used to measure the significance of the function [26].The significant GO terms were identified by P<0.05 and FDR <0.05.

### miRNA profiling and prediction of novel miRNAs and mRNA targets

The raw small RNA reads were processed with FastQC (http://www.bioinformatics. bbsrc.ac.uk/projects/download.html#) for quality control using the following criteria: i) remove reads with >30% bases with quality <20; ii) remove reads with lengths <15 bp; and iii) remove adaptor sequences. The small RNA reads were aligned to Sanger miRBase (version 21.0) and the bovine genome by BWA software (http://bio-bwa.sourceforge.net/). The EBseq algorithm was also employed to filter the DE miRNAs based on FC and FDR thresholds [20]. The DE miRNAs were identified according to the following criteria: (1) |log2(FC)|>1; and (2) FDR<0.05. Novel miRNAs were predicted with the miRDeep program, and the secondary structures of inverted repeats were predicted using RNAfold (http://www.rnasoft.ca/download.html) [27]. After predicting the novel miRNAs, the remaining reads were subjected to Blast searches against rat, mouse and human miRNA databases (version 21.0). MiRanda tools (http://www.microrna.org/ microrna/getDownloads.do) were used to predict the potential targets of the DE miRNAs because this method can identify a significant number of experimentally determined non-canonical and non-conserved sites [28].

### Construction of miRNA target network and molecular interaction network

DE mRNAs and miRNAs were integrated to determine whether the expression levels of each miRNA and its target were negatively correlated by using a previously published methods [29]. The relationships between the miRNAs and the genes were counted based on their DE values according to the interactions between the miRNAs and the genes in the Sanger miRNAs database, thereby generating a miRNAs target network. The adjacency matrix of the miRNAs and genes A = [ai, j] was produced by attributing the relationships among the genes and the miRNAs, with ai, j representing the relationship between the weight of gene i and miRNAs j. In the microRNA-target network, a circle with different colors represents a gene or microRNA, and their relationship is represented by one edge. The center of the network is represented as degrees, which indicate the contribution of one miRNA to the surrounding genes or the contribution of one gene to the surrounding miRNAs.

Known DE bovine miRNAs were classified into family based on sequence similarity in the seed region, using TargetScan program (http://www.targetscan.org), and the family members intend to share the similar biological and medicine function [30–33]. The homologous miRNAs and their negatively correlated DE target genes were used for further analysis. The Ingenuity Pathway Analysis (IPA, Ingenuity® Systems—www.ingenuity.com) was used to construct a molecular interaction network. IPA is a highly convenient software application that can help biologists to classify the pathways, molecular networks and functions most relevant to genes of interest or experimental datasets [33–38].

### qRT-PCR analysis

qRT-PCR analyses were performed by using an Applied Biosystems 7500 Fast Real-Time PCR System (Applied Biosystems, Foster City, CA, USA). Total RNA from IMF tissues was isolated using the TRIzol^®^ Reagent (Invitrogen, Carlsbad, CA, USA); cDNAs were synthesized using a TaKaRa^®^ PrimeScript^TM^ RT reagent kit with a gDNA Eraser; and qRT-PCR analyses were carried by using TaKaRa^®^ SYBR^®^ Premix Ex TaqII (Tli RNaseH Plus). mRNA primers were designed using Primer 5.0 software (Primer-E Ltd., Plymouth, UK). The first-strand cDNA of miRNAs was synthesized using the TIANGEN^®^ miRcute miRNA First-Strand cDNA Synthesis kit.

The design of miRNA primers and the expression of miRNAs were carried out using the TIANGEN^®^ miRcute miRNA qPCR Detection kit (SYBR Green). Measurements were performed in triplicate. For validating the RNA-seq data, the geometrical mean of *β-actin* and *Rps18* (for mRNA) or *Rps18 and U6* (for miRNA) were used as a control, whereas *β-actin* (for mRNA) or *Rps18* (for miRNA) were used as reference genes for detecting gene expression in cells. All the primers were available on request (S1 Table).

### Isolation of primary bovine pre-adipocytes, cell culture and transfection

Healthy calves (1 day of age) were used for Isolating primary bovine pre-adipocytes. Primary bovine pre-adipocyte isolation and bovine pre-adipocyte differentiation were done as previously described [39]. Cells were cultured on 12- or 6-well plates (Costar, Corning Inc, Corning, NY). Chemically synthetic *bta*-*let-7i* and *bta-miR-2305* mimics or inhibitor and the negative control (purchased from Ribobio, Guangzhou, China) were transfected into 293A or primary pre-adipocyte cells using a standard reverse transfection protocol at 50 nM. Briefly, X-tremeGene HP DNA Transfection Reagent (Roche, Mannheim, Germany) was diluted in Opti-MEM (Life Technologies, Carlsbad, CA, USA) and added to the miRNAs; the cells were seeded 30 min later. Each transfection was done in triplicate, and RNA and protein were extracted 6 d after inducing differentiation for subsequent experiments.

### Plasmid construction and luciferase reporter assays

Chemically synthetic candidate target sequences containing wild-type and mutant seed regions (purchased from the Hanbio Technology Co. Ltd, Shanghai, China) were cloned to psiCHECK-2 to construct wild-type and mutant reporter plasmids, respectively. The bta-miRNA mimics (500 ng) and negative control were co-transfected with 500 ng candidate target gene-containing wild-type and mutant psiCHECK-2 vectors into the 293A cells. Cells were collected 48 h later to assay luciferase activity.

### Oil Red O staining and triglyceride content

TG content in the adipocytes was determined using the Bovine TGs ELISA Kit (Jining, Jining Inc, Shanghai, China). For Oil Red O staining, the cells were washed with PBS, fixed in 4% polyformaldehyde for 30 min, and washed and stained with 60% Oil Red O solution (solvent: isopropanol, 0.5 g Oil Red powder/100 ml) for 30 min in the dark. Stained cells were washed in PBS before being examined.

### Immunostaining

Cell culture immunofluorescence was observed as described by Clark *et al*. [40]. Anti-perilipin A (1:1000) and donkey anti-rabbit IgG H&L (Alexa Fluor® 647) (1:2000) antibodies were used (both from Abcam Inc., Cambridge, MA, USA).

### Statistical analysis

All numerical data are expressed as the mean ± SEM. Statistical differences among groups were analyzed by one-way analysis of variance with a post-hoc test to determine group differences in the study parameters. All statistical analysis were performed with SPSS 16.0 software (SPSS, Chicago, IL, USA). Statistical differences between two groups were determined by the Student’s *t* test. P < 0.05 was considered statistically significant.

## Results

### IMF content, serum testosterone levels and serum lipids

**Fig 1:**
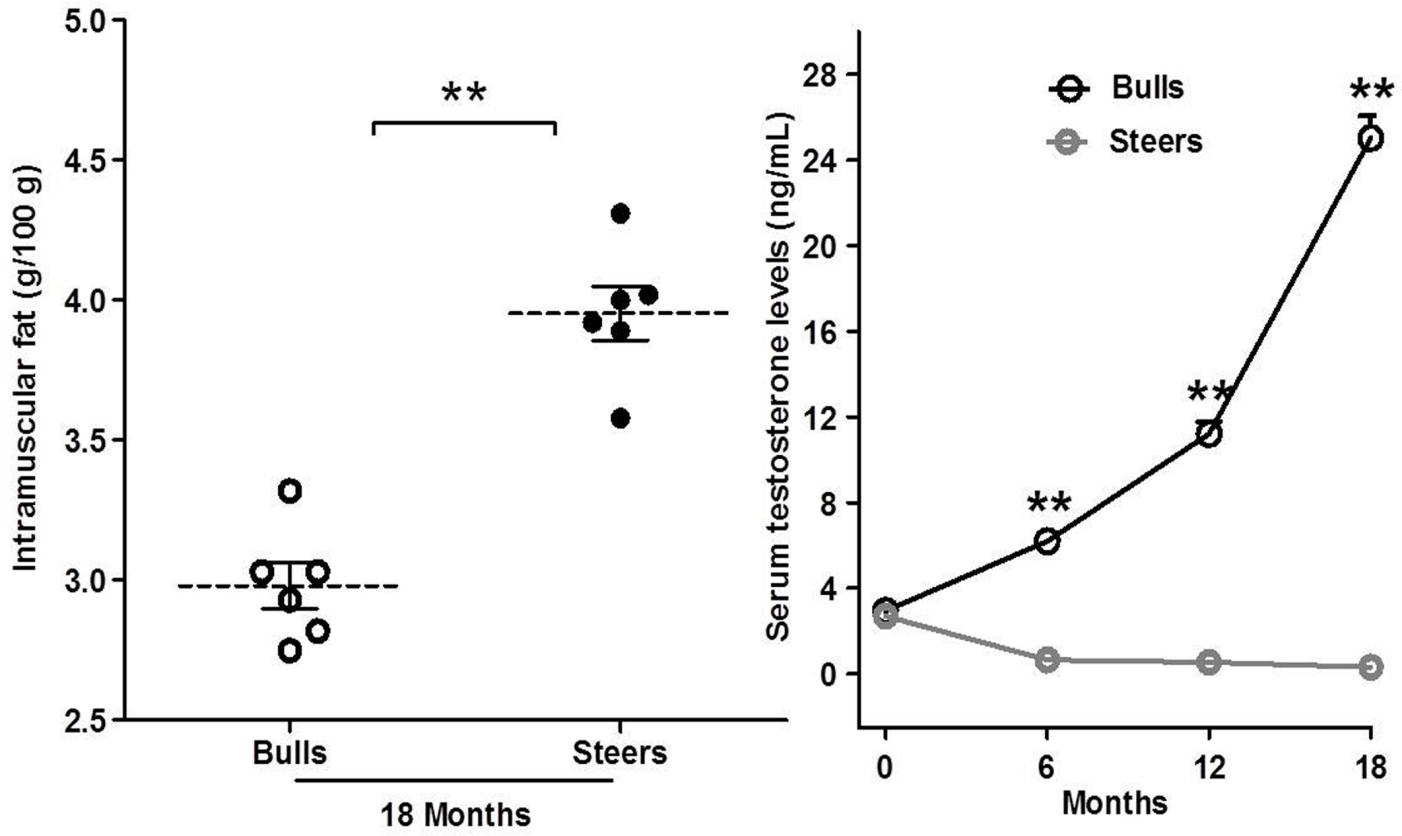
Effects of castration on intramuscular fat (IMF) and serum testosterone concentration. A: IMF content. B: Serum testosterone concentration. The data are expressed as the mean ± SEM, n = 6 per group. *P<0.05 and **P<0.01.

The steer group had a significantly higher IMF content than the bulls group at 18 months post-castration (n = 6; P<0.01; Fig 1A), whereas the steers had significantly lower serum testosterone levels than the bulls at 6 (0.64 ± 0.01 vs. 6.19 ± 0.38 ng/mL), 12 (0.54 ± 0.01 vs. 11.20 ± 0.57 ng/mL) and 18 months (0.30 ± 0.01 vs. 25.03 ± 0.85 ng/mL) post-castration (P<0.01; Fig 1B). Testosterone deficiency caused by castration significantly decreased serum testosterone levels and significantly increased in the IMF contents, as anticipated.

**Fig 2:**
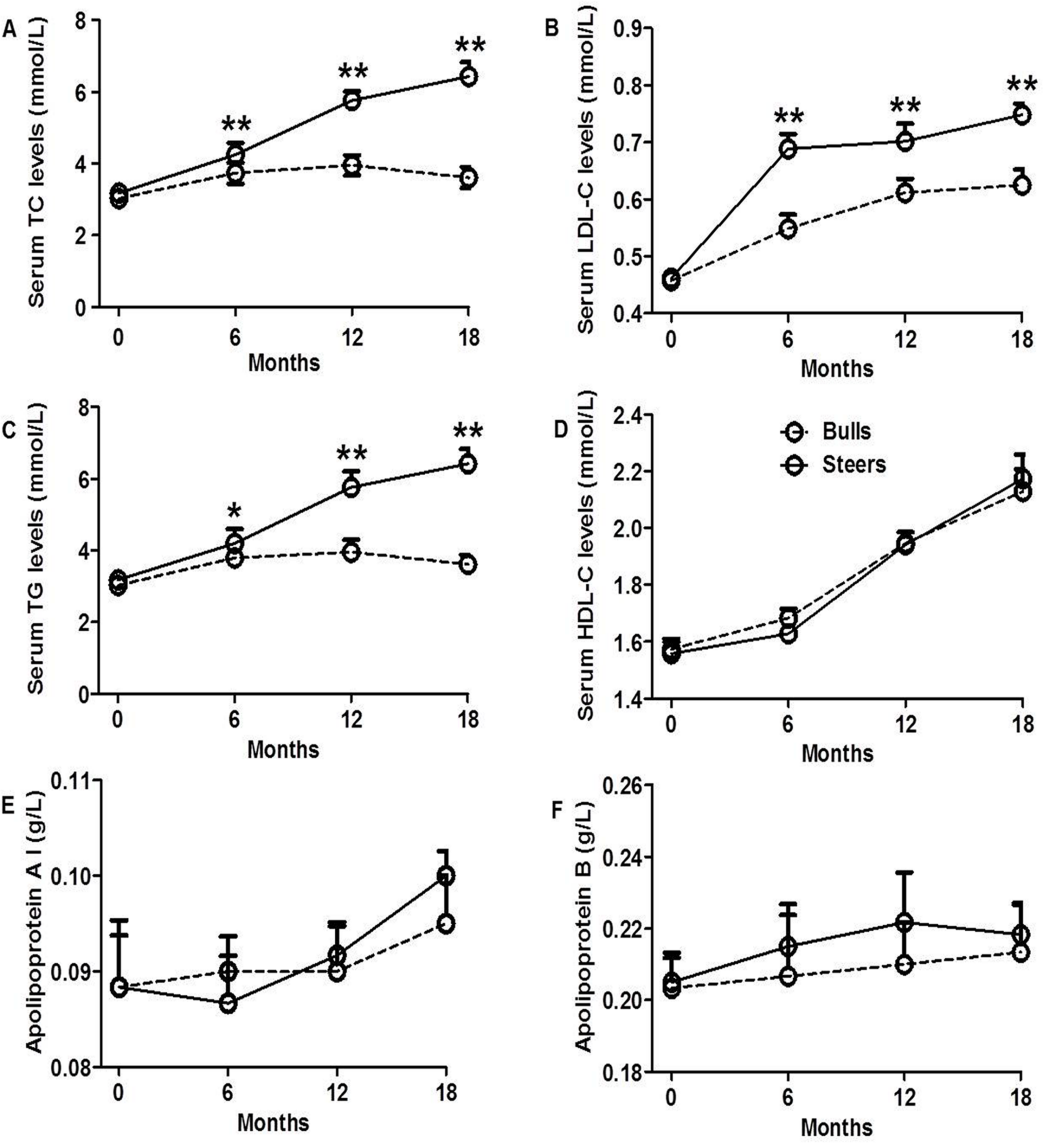
Effects of castration on serum lipids level. A: Serum total cholesterol (TC) levels. B: Low-density lipoprotein cholesterol (LDL-C) levels. C: High-density lipoprotein cholesterol (HDL-C) levels. D: Triglyceride (TGs) levels. E: Apolipoprotein A levels. F: Apolipoprotein B levels. The data are expressed as the mean ± SEM, n = 3 per group. *P<0.05 and **P<0.01.

Serum lipids parameters are shown in Fig 2A, 2B, 2C, 2D, 2E and 2F. Steers had higher TC levels than bulls at 6, 12 and 18 months after castration (P<0.05; Fig 2A), and serum LDL-C and TGs levels followed a pattern similar in both groups (Fig 2B and 2C). However, testosterone deficiency after castration did not significantly influence serum HDL-C, apolipoprotein A (apoAI), and apolipoprotein B (apoB) levels (Fig 2D, 2E and 2F).

### Differential expression of mRNAs in IMF from steers and bulls

**Fig 3:**
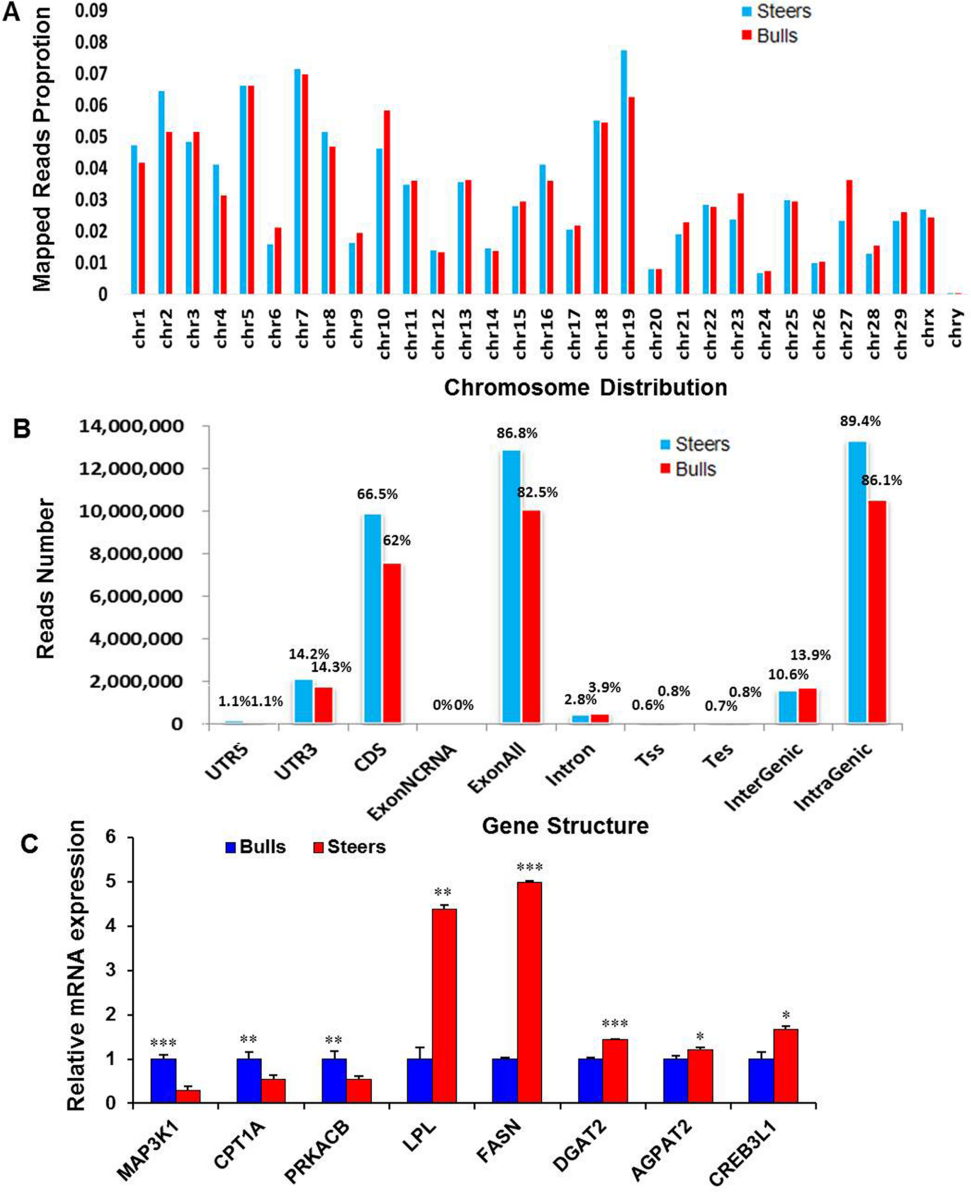
Characterization of the RNA-seq mapped reads in the intramuscular fat (IMF) tissue from steers and bulls. A: Read distributions of each chromosome. B: Regional distribution of the mapped reads. Approximately 66.5% (steers) and 62% (bulls) of the reads mapped to coding sequence (CDS) regions. C: qRT-PCR verification of selected genes expression in steers and bulls. The relative expression levels of these genes were normalized to *β-actin* and *Rps18*. * P<0.05, **P<0.01, ***P<0.001, independent samples t-test. n = 3 replicates per group. Error bars represent the mean ± SEM.

Following mRNA sequencing, the raw sequence yielded ~2.5 and 2.1 gigabases (GB) of data for the steers and bulls libraries, respectively. Approximately 84% of the total raw reads were uniquely mapped to bovine genome sequences in the steers and bulls samples (S2 Table). The mapped reads in the both groups were consistently distributed on the chromosomes (Fig 3A). Among the uniquely mapped reads, >62% were aligned to coding sequence (CDS) regions, 2.8% to intron regions, 15% to untranslated region (UTR) regions, 10% to intergenic regions, and 1% to a transcriptional start site (TSS) or transcription end site (TES) (Fig 3B). After annotation, 25,444 transcripts were annotated with known functions. Among them, expression of 580 genes was upregulated and 1,120 genes downregulated (|log2 (FC)|>1and FDR <0.05) in steers IMF compared with bulls IMF (S3 Table). Expression of some DE genes was confirmed by qRT-PCR (Fig 3C). We found that the expression of *mitogen-activated protein kinase kinase kinase 1* (*MAP3K1*), *carnitine palmitoyltransferase 1A* (*CPT1A*) and *protein kinase cAMP-dependent catalytic beta* (*PRKACB*) was downregulated in steers; in contrast, *lipoprotein lipase* (*LPL*), *FASN*, *diacylglycerol O-acyltransferase 2* (*DGAT2*), *1-acyl-sn-glycerol-3-phosphate acyltransferase* (*AGPAT2*) and *Cyclic AMP-responsive element-binding protein 3-like protein 1* (*CREB3L1*) were significantly upregulated in steers. qRT-PCR analysis largely confirmed the RNA-seq data.

**Fig 4:**
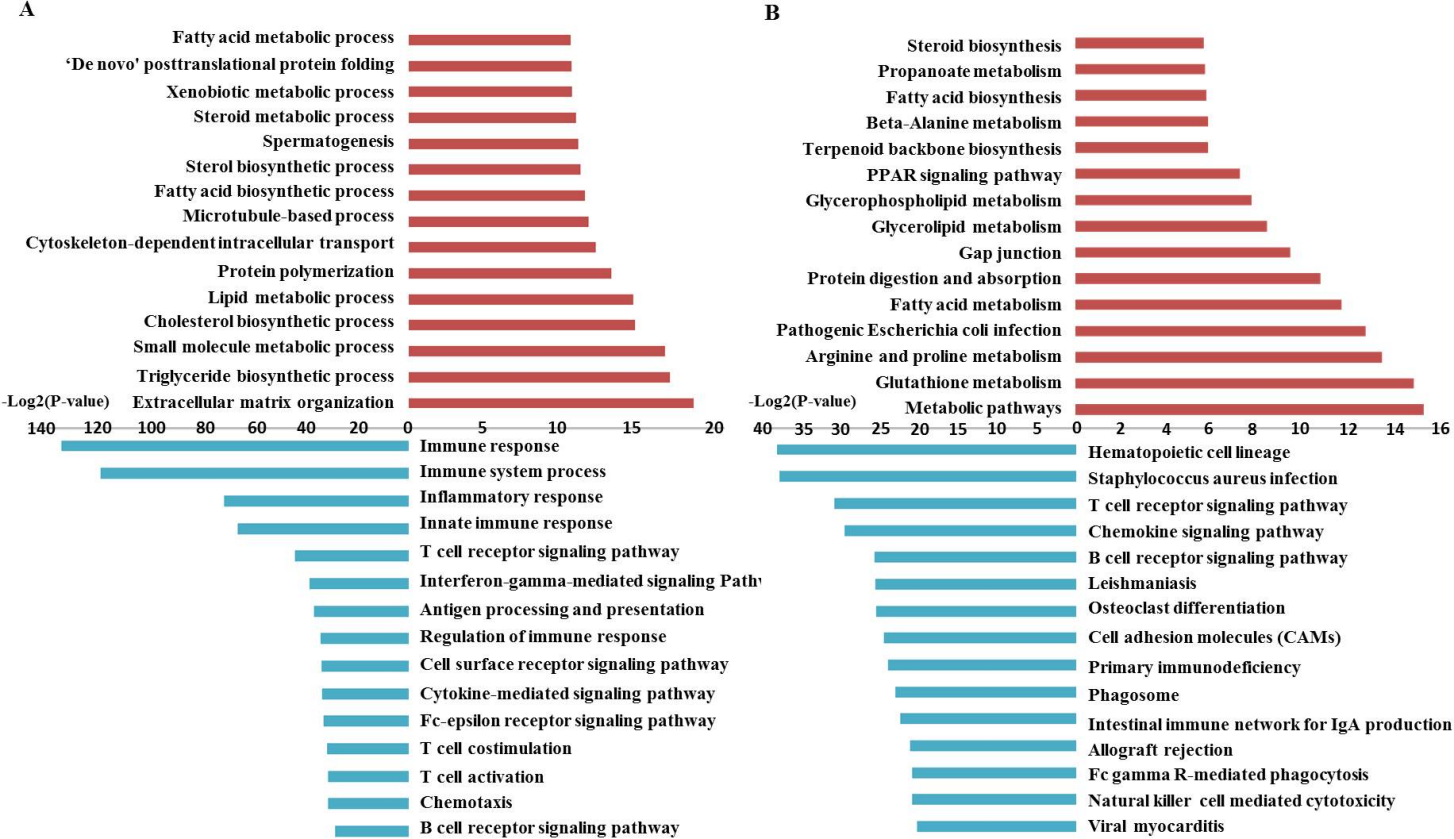
Gene ontology (GO) and pathway analysis of differentially expressed (DE) genes. A: GO analysis of DE genes between steers and bulls; B: Pathway analysis was conducted for DE genes between steers and bulls. The red color shows the upregulated DE genes and blue the downregulated DE genes.

To annotate the 1,700 DE genes related to IMF regulation, the GO and KEGG pathways were analyzed for the upregulated and downregulated DE genes, respectively. Upregulated genes were mainly related to extracellular matrix organization, triglyceride biosynthetic process, small molecule metabolic process, cholesterol biosynthesis, lipid metabolism, and fatty acid biosynthesis. The GO terms of the downregulated genes included immune response, intracellular signal transduction and cell adhesion (Fig 4A, S4 and S5 Tables). Pathway analysis showed that the upregulated genes were associated with metabolic pathways, glycerolipid metabolism, fatty acid metabolism, glycerolipid and glycerophospholipid metabolism, fatty acid biosynthesis and peroxisome proliferator-activated receptor (PPAR) signaling pathway, and that decreased gene expression was related to hematopoietic cell lineage, T cell receptor signaling pathway, cell adhesion molecules (CAMs) and some hormone signaling pathways (Fig 4B, S6 and S7 Tables). From these results, we conclude in general that IMF tissues significantly increased lipid biosynthesis in response to castration, including activation of triglycerides, and cholesterol and fatty acid biosynthesis. Concurrently, cellular activity related to immune response and signal transduction in IMF tissues was inhibited by castration. This is consistent with the characteristics of steers, which have higher IMF contents and serum lipids levels, as well as lower serum testosterone levels (Figs 1 and 2).

### Analysis of miRNAs expression patterns in IMF from steers and bulls

**Fig 5:**
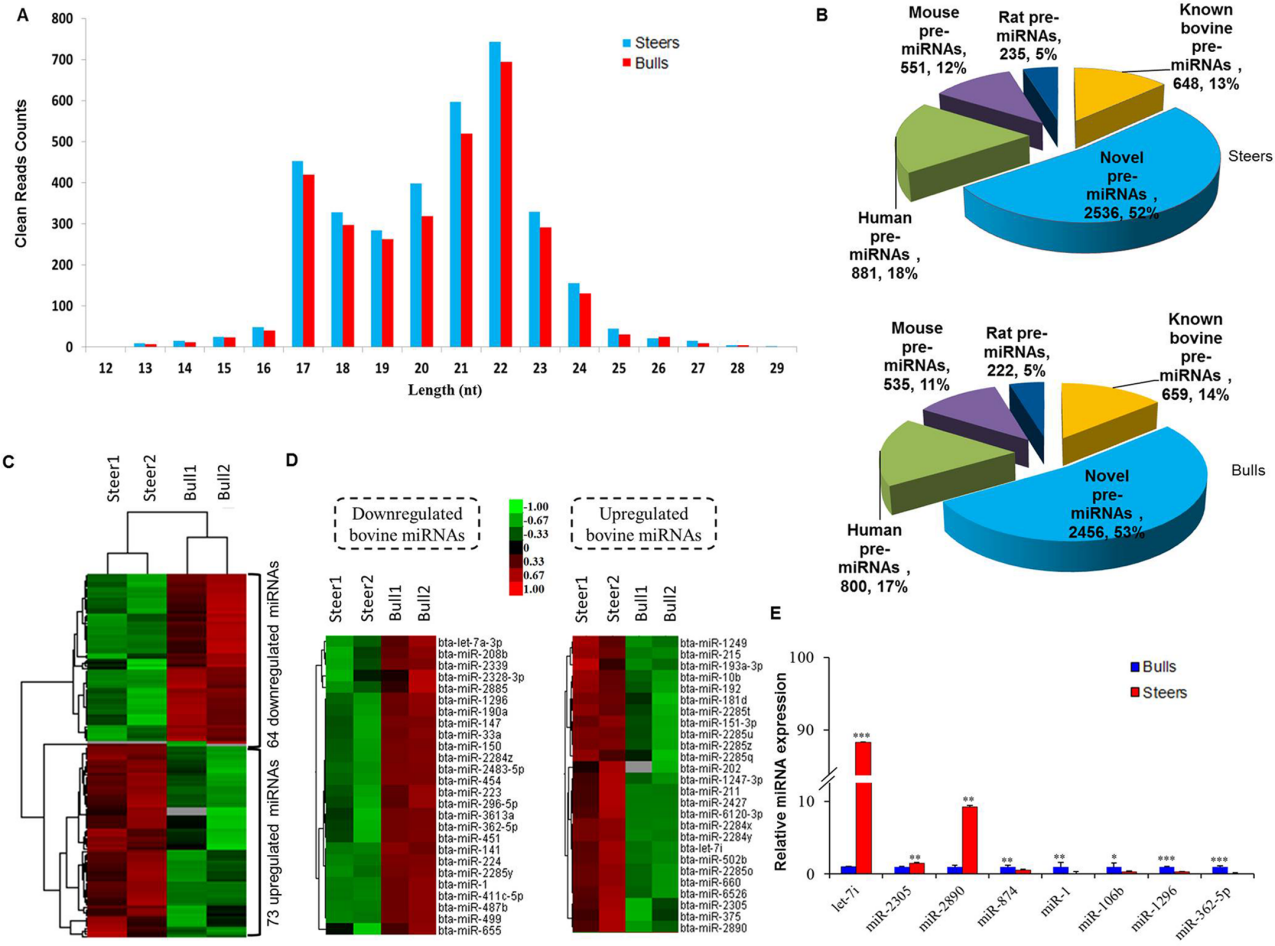
Differential miRNAs expression between steers and bulls. A: Length distribution of the clean reads. Most of the reads had a length of 21–22 nt. B: Number and percentage of identified miRNAs. A total of 5,530 unique miRNAs were identified. C: The Global miRNA expression profile of the IMF tissue from steers and bulls was analyzed by RNA-seq analysis. The heat map shows the differentially expressed (DE) miRNA patterns (|log2 (FC)|>1and FDR <0.05). D: Upregulated (left) and downregulated (right) bovine miRNAs in the IMF tissue between steers and bulls are shown as a heat map; a total of 52 DE bovine known miRNAs have been identified. E: qRT-PCR verification of selected miRNAs expression in (C). Relative expression of these miRNAs was normalized to *β-actin* and *U6*. * P<0.05, **P<0.01, ***P<0.001, independent samples t-test. n = 3 replicates per group. Error bars represent the mean ± SEM.

In total, miRNA-seq yielded a total of 10,782,869–13,832,768 clean reads size from 12–29 nt for all 4 samples (S8 Table). By aligning the clean reads against the bovine genome sequences (Btau4.6.1), ~86% in each library were matched to the bovine genome. Size distribution of the clean reads was shown in Fig 5A. The number of 21–22 nt sequences was significantly greater than the number of shorter or longer sequences, consistent with the features of mature miRNAs.

A read was assigned to a miRNA by blasting against the non-miRNA databases. A total of 5,530 unique miRNAs, comprising 2,907 known pre-miRNAs and 2,623 novel pre-miRNAs, were identified. A total of 612 bovine pre-miRNAs overlapped between the steers and bulls groups. Another 648 and 659 known bovine pre-miRNAs genes were identified in the steers and bulls libraries (S9 Table). From the dataset, 2,536 and 2,456 novel bovine pre-miRNAs were predicted to be included in the steers and bulls libraries, respectively (S10 Table), and among the remaining reads that did not successfully predict novel miRNAs, 1,667 and 1,557 pre-miRNAs in the bulls and steers libraries, respectively, were homologous to human, rat and mouse genomes. The number and percentage of identified miRNAs are shown in Fig 5B.

MiRNAs are a kind of non-coding RNA implicated in the development of adipose tissue after castration [10–13]. Therefore, miRNAs might be important in generating high IMF deposition through modulating the expression of targeting genes. Given that the transcriptional levels of thousands of genes were altered between steers and bulls IMF tissues, changes in the expression of a proportion of genes should be elicited by DE miRNAs, which were detected by miRNAs expression profiling. Among the detected miRNAs, 137 DE genes comprising 52 bovine known genes, 72 predicted novel genes and 13 homologs genes in the human, mouse and rat databases were identified in bulls and steers cattle (|log2FC| ≥1, FDR <0.05). Compared with bulls, 26 known bovine miRNAs were upregulated and 26 bovine known miRNAs were decreased (|log2FC| ≥1, FDR <0.05) in steers (Fig 5C and 5D).

Regarding qRT-PCR used validate relative gene expression of the 8 selected miRNAs, we showed in Fig 5E that *miR-let-7i*, *miR-2305* and *miR-2890* were upregulated and the expression of *miR-874*, *miR-1*, *miR-106b*, *miR-1296* and *miR-362-5p* decreased in steers. The tendency for changes in expression was consistent between microRNA-seq and qRT-PCR results. These DE miRNAs might be associated with the development of IMF tissues in steers.

### Target predictions of DE miRNAs and integrative analysis

**Fig 6:**
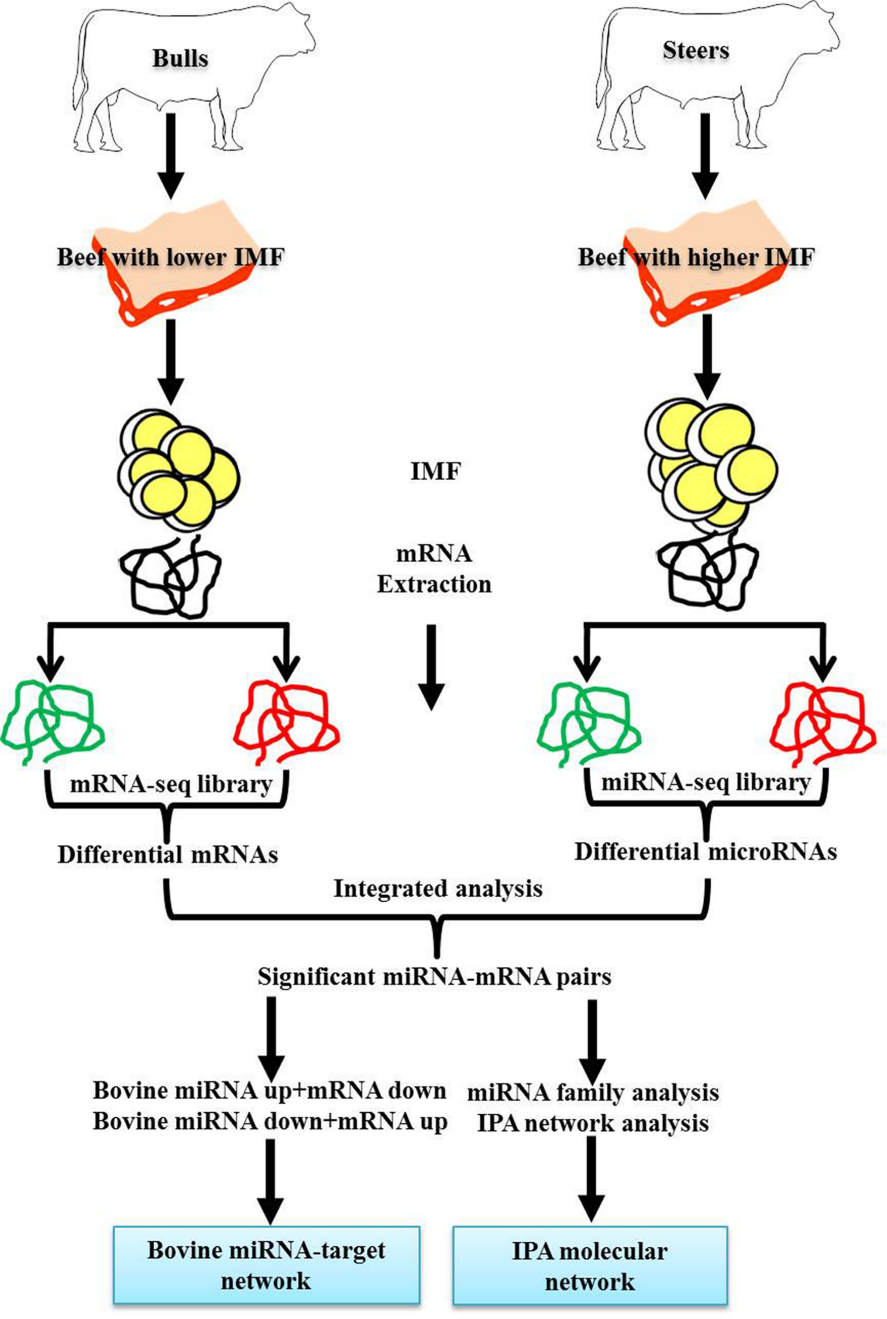
Outlines for establishing miRNA target networks and molecular network associated with intramuscular fat (IMF) regulation following castration.

**Fig 7:**
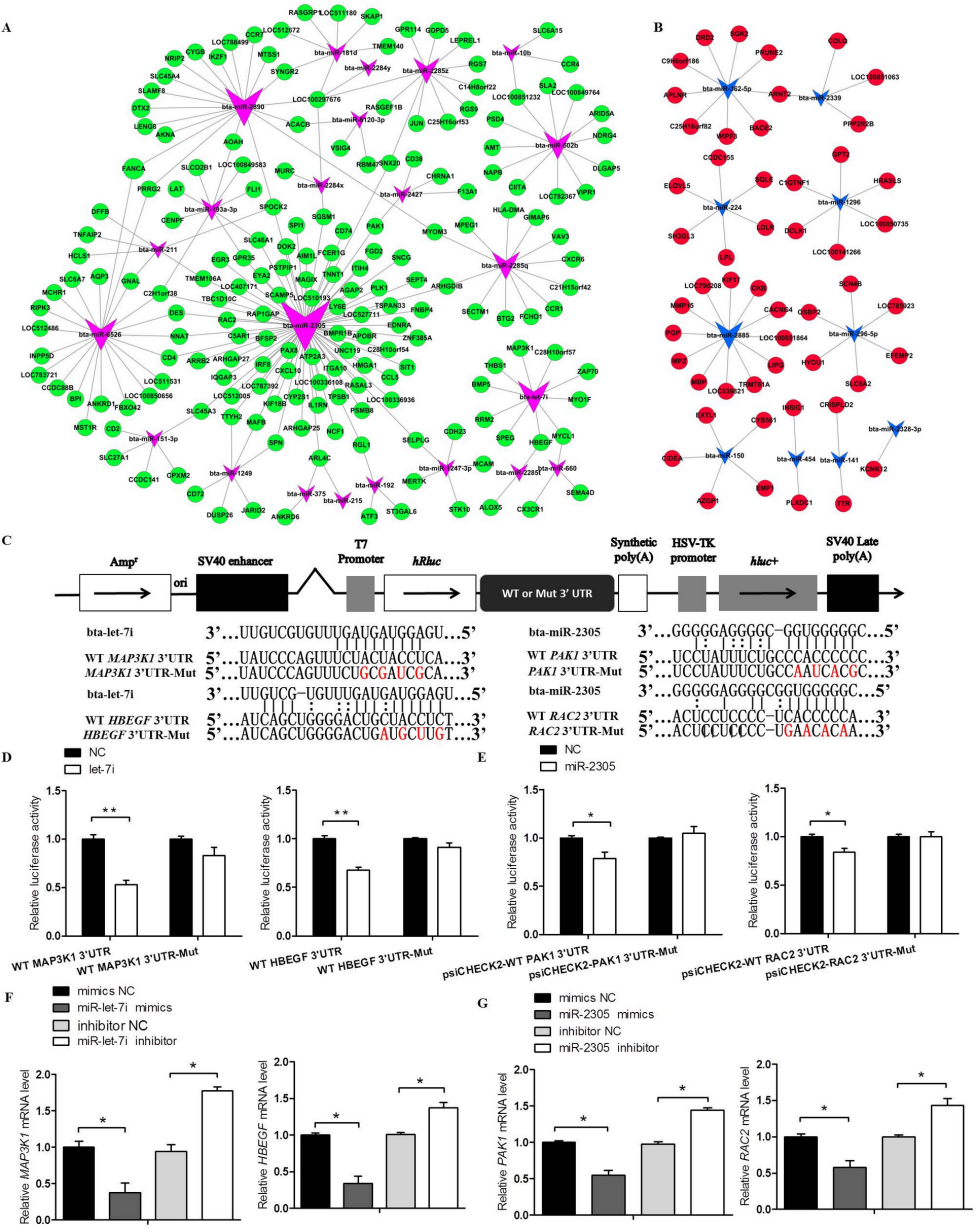
The microRNA-target network in intramuscular fat (IMF) tissues from steers. A: An up microRNA-down target network was constructed by integrating the expression change of predicted genes in IMF tissues from steers and bulls and functional annotations of these genes. B: A down microRNA-up target network was constructed by integrating the expression change of predicted genes in IMF tissues from steers and bulls, and functional annotations of these genes. C: Schematic diagram illustrating the design of the WT *MAP3K1*, *HBEGF*, *PAK1* and *RAC2* 3’UTR luciferase reporters (WT 3’UTR) or the corresponding site-directed mutant gene 3’UTR (3’UTR-Mut). D, E: Effect of *bta*-*let-7i*–mimic or *bta-miR-2305* mimic and its mimic-NC on luciferase activity in 293A cells transfected with either the WT 3’UTR or 3’UTR-Mut reporter. F: Effect of *bta-let-7i*-mimics, mimic-NC or *bta-let-7i*-inhibitor, inhibitor-NC on *MAP3K1* or *HBEGF* mRNA levels in bovine pre-adipocytes. G: Effect of *bta-miR-2305*-mimics, mimic-NC or *bta-miR-2305*-inhibitor, inhibitor-NC on *PAK1* and *RAC2* mRNA levels in bovine pre-adipocytes. The data are presented as the mean ± SEM. *P<0.05, **P<0.01. One-way ANOVA with a post-hoc test was used, and statistical differences between the two groups were determined by the independent samples t-test.

To predict the target genes of the DE bovine miRNAs, they were computational analysed with the Miranda program (www.miranda.org.uk). Through the research strategy described in Fig 6, integrative analysis of mRNA and microRNA profiles was used. A total of 7,418 putative targets of 137 DE pre-miRNAs are shown by applying the criteria of a free binding energy of ≤20 and a score of ≥150 (Energy ≤-20, Score ≥150). Moreover, if any of DE miRNAs and DE mRNAs were negatively correlated, we considered that it was highly probable that the gene identified was a true target of the miRNA being analyzed. In total, 951 DE miRNA-mRNA pairs including 103 DE miRNAs and 535 DE mRNAs were negatively correlated (Energy ≤-20, Score ≥150) (S11 Table). For most miRNAs, multiple targets were identified, but some of the miRNAs only targeted only one target gene. Furthermore, a single gene was potentially targeted by several miRNAs. To illustrate these findings more clearly, functional network of the known bovine miRNAs-targets pairs were constructed using cytoscape v2.8.2 (Fig 7A and 7B).

The dual luciferase reporter system was then used to validate the target genes described in Fig 7. To determine whether *bta-let-7i* and *bta-miR-2305* can directly target their predicted target genes, including *MAP3K1*, *proheparin-binding epidermal growth factor-like growth factor* (*HBEGF*), *serine/threonine-protein kinase* (*PAK1)* and *Ras-related C3 botulinum toxin substrate 2 (RAC2*), we designed luciferase reporter constructs that included either the wild- or mutant-type 3’-UTR of *MAP3K1*, *HBEGF*, *PAK1* and *RAC2* (Fig 7C). Of note, co-transfection with *bta-let-7i* and *bta-miR-2305* significantly suppressed luciferase activity (Fig 7D and 7E), and wild-type *MAP3K1*, *HBEGF*, *PAK1*, *RAC2* groups had approximately 48% (P<0.01), 33% (P<0.01), 22% (P<0.05) and 17% (P<0.05) less luciferase activity, respectively, than the corresponding NC groups. However, there was no discernable difference in luciferase reporter expression after independent transfection of wild-type and mutant constructs into bovine pre-adipocytes (Fig 7D and 7E). The effects of overexpression or suppression of *bta*-*let-7i* or *bta-miR-2305* on the levels of mRNA expression of endogenous *MAP3K1*, *HBEGF*, *PAK1* and *RAC2* were further examined. Bovine pre-adipocytes were transfected with *bta*-*let-7i* or *bta-miR-2305* mimics, inhibitor and NC. The qRT-PCR results showed that *bta*-*let-7i* or *bta-miR-2305* could markedly affect the expression of *MAP3K1*, *HBEGF*, *PAK1* and *RAC2* mRNA (P<0.05; Fig 7F and 7G). These results demonstrated that *MAP3K1* and *HBEGF* are targets of *bta-let-7i*, whereas *PAK1* and *RAC2* are targets of *bta-miR-2305*. This verification provides strong support for the construction of the known bovine miRNAs-mRNA pairs by negative correlation analysis. These DE miRNAs and their putative targets might be associated with the development of IMF tissues in CM cattle.

### Molecular network in bovine IMF potentially regulates IMF following bull castration

To generate a potential network of interacting DE genes and miRNAs in steers IMF that might be involve in regulating IMF deposition in steers cattle, miRNA family identification and IPA gene interaction network were analyzed. Among the 103 DE miRNAs screened by negative correlation analysis (|log2(FC)| ≥1, FDR <0.05; Energy ≤-20, Score ≥150), 23DE known bovine miRNAs were classified into miRNA families with sequence similarity in the seed regions and biological functions [30–33]. A total of 16 DE known bovine miRNAs showed sequence homology with human miRNAs (Table 1).

**Table 1:** List of 23 DE bovine miRNAs in IMF tissues between steers and bulls and the identification results of miRNA family.

Bovine miRNA	Log2FC (steers /bulls)	FDR	Mature Sequence	Seed Region of miRNA Family (3’-5’)	Human miRNA
bta-miR-6526	4.758	4.37E-04	tcctgtgcctcgaatgggtatg	CCUGUGC	hsa-miR-1914-5p
bta-miR-2890	4.591	6.33E-03	gccccggccgctcccggcct	CCCCGGC	hsa-miR-4707-5p
bta-miR-2305	3.245	3.75E-04	cgggggtggcggggaggggg	GGGGGUG	hsa-miR-6752-5p
bta-miR-193a-3p	1.887	0.04	aactggcctacaaagtcccagt	ACUGGCC	hsa-miR-193a-3p
bta-miR-192	1.436	4.96E-06	ctgacctatgaattgacagccag	UGACCUA	hsa-miR-192-5p
bta-miR-1247-3p	1.311	2.66E-03	cgggaacgtcgggactggagc	GGGAACG	hsa-miR-1292-5p
bta-miR-151-3p	1.251	3.91E-05	ctagactgaagctccttgagg	UAGACUG	hsa-miR-151a-3p
bta-miR-6120-3p	1.152	3.65E-06	tatgttggacaacgtggatagc	AUGUUGG	hsa-miR-4781-3p
bta-let-7i	1.071	5.12E-05	tgaggtagtagtttgtgctgtt	GAGGUAG	hsa-let-7a-5p
bta-miR-141	-2.097	0.03	taacactgtctggtaaagatgg	AACACUG	hsa-miR-141-3p
bta-miR-224	-1.921	6.82E-04	caagtcactagtggttccgttta	AAGUCAC	hsa-miR-224-5p
bta-miR-454	-1.481	0.01	tagtgcaatattgcttatagggt	AGUGCAA	hsa-miR-130a-3p
bta-miR-362-5p	-1.422	0.01	aatccttggaacctaggtgtgagt	AUCCUUG	hsa-miR-362-5p
bta-miR-150	-1.117	0.04	tctcccaacccttgtaccagtgt	CUCCCAA	hsa-miR-150-5p
bta-miR-296-5p	-1.090	1.37E-03	gagggccccccccaatcct	AGGGCCC	hsa-miR-7160-3p
bta-miR-1296	-1.039	4.31E-03	ttagggccctggctccatctcc	UAGGGCC	hsa-miR-1296-5p
bta-miR-2427	2.341	2.56E-03	aggtcatttcaaagagggctg	GGUCAUU	/
bta-miR-2285z	1.406	2.83E-08	ccagaaagttcattcaggtcct	CAGAAAG	/
bta-miR-502b	1.398	5.20E-09	aatccacctgggcaaggattc	AUCCACC	/
bta-miR-2285t	1.192	4.93E-04	agaatctggatgaactttttgg	GAAUCUG	/
bta-miR-2285q	1.156	4.53E-02	aaggacctgaatgaactttctgg	AGGACCU	/
bta-miR-2483-5p	-1.869	7.83E-03	cgtcaaccatccagctgtttga	GUCAACC	/
bta-miR-2885	-1.083	0	cggcggcagcgccggggcg	GGCGGCA	/

Note: FC, Fold Change, FDR, False Discovery Rate. Differentially Expressed (DE) miRNAs were identified according to the following criteria: |log2(FC)|>1 and FDR<0.05.

**Fig 8:**
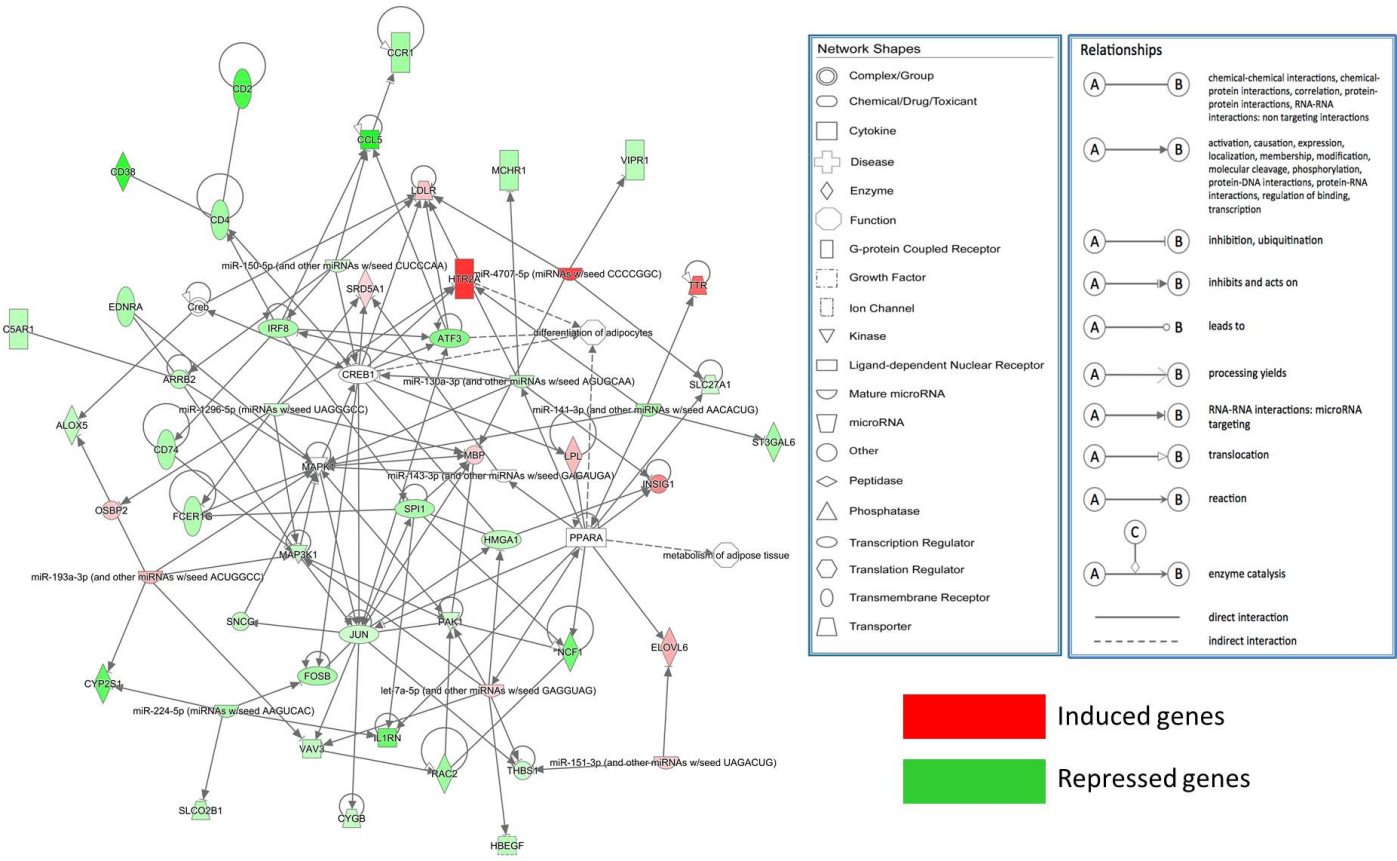
Molecular networks functionally associated with adipocyte differentiation and adipose tissues metabolism identified by Ingenuity Pathway Analysis (IPA).

These 16 DE miRNAs and their DE target genes (|log2(FC)| ≥1, FDR <0.05; Energy ≤-20, Score ≥150) were used for construct a gene interaction network in the IPA system. Fig 8 shows that only 9 DE miRNAs (*bta-let-7i*, *bta-miR-1296*, *bta-miR-141*, *bta-miR-150*, *bta-miR-151-3p*, *bta-miR-193a-3p*, *bta-miR-224*, *bta-miR-2890*, and *bta-miR-454*) and 42 DE target genes were observed. Using IPA software, biological function analysis of these target genes showed that the most important functions of this network consisted of differentiation of adipocytes and metabolism of adipose tissue (S12 Table). From this analysis, *bta-let-7i* in the molecular network was selected for further analysis.

### Function identification of miRNA in bovine IMF tissues

**Fig 9:**
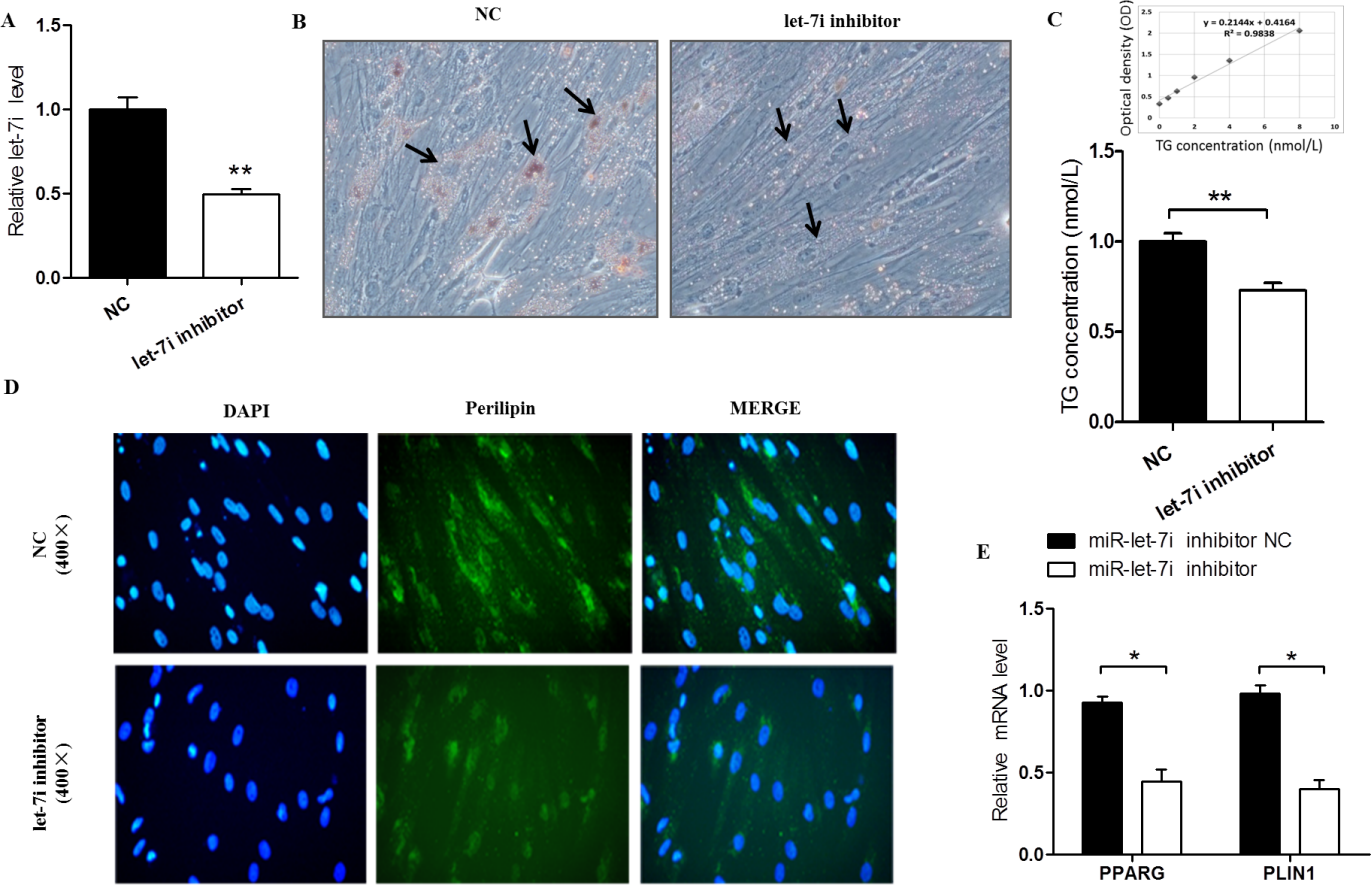
Function identification of miRNA in bovine intramuscular fat (IMF) tissues. A: Relative expression levels of *bta-let-7i* in the *bta-let-7i* inhibitor and inhibitor-NC groups. B: Lipid droplet formation in the inhibitor-NC and *bta-let-7i* inhibitor groups after differentiation induction (oil red O staining, 200×). The arrow indicates a differentiated adipocyte. C: Triglyceride (TGs) concentration showing the linear regression equation of the standard curve. D: Bovine pre-adipocytes were transfected with *bta-let-7i*-inhibitor and inhibitor-NC for 6 d and stained for adipocyte perilipin using anti-Perilipin A antibody (green) and DAPI (blue) (400×). E: qRT-PCR analysis measuring the relative level of adipogenic marker genes, including *PPARG* and *PLIN1*. Data are presented as the mean ± SEM. *P<0.05, **P<0.01. One-way ANOVA with a post-hoc test was used, and statistical differences between the 2 groups were determined by the independent samples t-test.

To investigate whether *bta-let-7i* are involved in bovine pre-adipocyte differentiation, the expression of *bta-let-7i* was suppressed. The *bta-let-7i* -inhibitor and its NC were transfected into pre-adipocytes, and *bta-let-7i* expression significantly downregulated (P<0.01, Fig 9A). After 6 d of differentiation, the number of lipid droplets was clearly decreased in the group of cells transfected with *bta-let-7i* inhibitor compared with the NC group (Fig 9B). Similarly, TGs concentration was significantly lower in the *bta-let-7i* inhibitor group (P<0.01, Fig 9C), and very few differentiated cells treated with *bta-let-7i* inhibitor expressed perilipin (Fig 9D), in which the expression levels of *peroxisome proliferator-activated receptor gamma* (*PPARG*) and *perilipin-1* (*PLIN1*) were markedly decreased (P<0.05, Fig 9F and 9G). These findings indicated that *bta-let-7i* could promote the differentiation of bovine pre-adipocytes, and also gives supports for the molecular network that helps direct further investigations of the miRNA-mediated physiological regulation in IMF tissue in cattle.

## Discussion

The content of IMF is closely associated with the meat quality of beef, which is one of the most important economic traits of beef production. In studying the effects of castration on the development of IMF deposition, steers had higher IMF contents and plasma lipid levels, including those of TC, LDL-C, and TGs, than the bulls group. However, testosterone levels following castration were clearly lower in the steers than the bulls. These results are reinforced by the meta-analysis performed by Whitsel *et al*. [41], who showed that the administration of intramuscular testosterone esters to hypogonadal men is associated with a small dose-dependent reduction of HDL-C concentrations and a decrease in TC and LDL-C in plasma. In orchidectomized mice fed a high-fat diet, Senmaru *et al*. [42] reported that testosterone deficiency could induce a markedly decreased serum TG level and increased LDL-C [43], which is consistent with our findings. Indeed, a common observation in several previous studies in pig and cattle has been a causal relationship between the accumulation of excess fat and low testosterone levels, regardless of where the fat deposits are located [5–7]. The possible reason of increased plasma lipids and IMF accumulation in steers may be that testosterone deficiency following castration diminishes the physiological effects of this androgen, such as reducing plasma lipids, increasing lipolysis by adipocytes and stimulation of androgen receptors [43]. However; others have demonstrated inconsistent results in castrated rodents [44]. The different effects of androgen deficiency on adiposity in these different species, including humans, could be explained by species-specific differences in the expression of genes involved in essential metabolic functions.

Little is known regarding the molecular mechanisms underlying the castration-induced promotion of IMF deposition in cattle. We have compared mRNAs and miRNAs to identify transcriptomic differences that may contribute to an increased IMF of cattle after castration using RNA-seq technology. To our knowledge, our work is the first to focus on IMF tissues to explore the mechanism of bovine IMF deposition after castration using the RNA-seq approach. By analyzing these DE genes and miRNAs, a microRNA-target network and an IPA molecular interaction network associated with lipogenesis and lipid metabolism has been established. The identified network might represent those regulations from miRNAs and mRNAs that underlie the development of IMF deposition after castration of bulls.

First, GO and KEGG pathway analyses showed that expression of genes involved in lipid metabolism, triglyceride and cholesterol biosynthetic process, fatty acid transportation and fatty acid biosynthetic process were differentially expressed in the steers compared with the bulls, which included glycolysis and pyruvate oxidation gene, such as *solute carrier family 25 member 1* (*SLC25A1*), NADPH generation genes such as *glucose-6-phosphate 1-dehydrogenase* (*G6PD*), fatty acid synthesis genes, such as *FASN*, *elongation of very long chain fatty acids protein 5* (*ELOVL5*), *elongation of very long chain fatty acids protein 6* (*ELOVL6*) and *17β-hydroxysteroid dehydrogenase isoform* (*HSD17B12*), fatty acid transportation genes, such as *Acyl-CoA-binding protein* (*DBI*), *LPL*, *long-chain-fatty-acid-CoA ligase1* (*ACSBG1*), *long-chain-fatty-acid-CoA ligase* (*ACSBG2*) *and Acyl-CoA synthetase long-chain family member 3* (*ACSL3*), and TGs synthesis genes, such as *glycerol-3-phosphate dehydrogenase* (*GPD1*), *glycerol-3-phosphate acyltransferase* (*GPAM*), *low-density lipoprotein receptor* (*LDLR*), *AGPAT2* and *DGAT2*. In contrast, fatty acid oxidation genes, such as *CPT1A*, *acetyl-coenzyme A carboxylase beta* (*ACACB*), and *enoyl-CoA hydratase* (*ECHS1*), were downregulated in the steers. TGs are major lipids that accumulate within myofibers and intramuscular adipocytes during IMF deposition [45]. The balance of synthesis and degradation of TGs including TGs biosynthesis, fatty acid mobilization, fatty acid transportation and oxidation determined the extent of fat accumulation in skeletal muscle [46]. By profiling hepatic gene expression by RNA-Seq in high-cholesterol-fed intact male pigs, castrated malepigs and castrated male pigs given testosterone replacement, Cai *et al*. [5] found that genes involved in fatty acid oxidation were downregulated in the liver tissues of castrated malepigs, whereas expression of fat synthesis genes increased [5]. In IMF tissues from the fatty pigs, higher expression levels of lipogenesis and fatty acid transportation genes occurred, whereas lipolysis genes were less expressed compared with lean pigs [47]. In *longissimus dorsi* muscle from castrated bulls, Jeong *et al*. [48] reported that castration shifts transcription of lipid metabolism genes, favoring IMF deposition by increasing adipogenesis, lipogenesis and triglyceride synthesis [7]. These results are in line with our findings in IMF tissues of steers. To date, few studies have explored the effects of castration on enzymes involved in TGs synthesis in adipocyte tissue [42, 48–50]. Jeong *et al*. [48] also reported that the mRNA level of *GPAM* in the *longissimus dorsi* muscle of steer is highly correlated with bovine IMF content (r = 0.74; P<0.001). Testosterone-deficient mice also had markedly increased *DGAT2* expression [42]. Results obtained in diacylglycerol:acyl-CoA acyltransferase (DGAT) deficient mice and 3T3-L1 cells overexpressing *DGAT2* have indicated the importance of this enzyme in energy production and fat accumulation [49,50]. In addition, *CPT1A*, which encodes a primary enzyme regulated in the overall mitochondrial fatty acid oxidation process, and *ACACB*, which coordinates the acute regulation of fatty acid oxidation along with *carnitine palmitoyltransferase1* (*CPT1*) and *malonyl-CoA decarboxylase* (*MCD*), were both downregulated in steers [51]. Together these findings suggest that the molecular mechanism of improved IMF in steers might be related to a higher capacity of lipogenesis and lower capacity of lipolysis directly induced by testosterone deficiency due to castration.

Our study also showed that most of the genes involved in the hormone-related signal transduction pathways, such as gonadotropin-releasing hormone (GnRH), estrogen, oxytocin and thyroid signaling pathways, were downregulated in the IMF of steers following castration, such as *transcription factor AP-1* (*JUN*), *MAP3K1*, *RAC2*, *phosphoinositide-3-kinase gamma polypeptide* (*PIK3CG*), *phosphatidylinositol 3-kinase*, *catalytic*, *beta polypeptide* (*PIK3CD*), *phosphoinositide 3-kinase regulatory subunit 5* (*PIK3R5*) and *receptor protein-tyrosine kinase* (*KIT*). Adipose tissue metabolism or pre-adipocyte proliferation and differentiation could be affected by gonadal steroid or peptide hormones, such as testosterone, estrogen, oxytocin and thyroid hormones [52–55]. Data from 3T3-L1 cells showed that dehydroepiandrosterone (DHEA) and 17β-estradiol directly influence the development of adipose tissue by reducing pre-adipocyte proliferation and differentiation [53]. Other studies have similarly demonstrated that DHEA induces anti-mitogenic effects when administered to pre-adipocytes from rats and pigs [56]. As regards thyroid, the findings from Oppenheimer *et al*. [55] showed that thyroid hormone only induces early increase in lipogenesis that serves to maintain fat stores, and then augments lipolysis in the rat. These findings suggest that the improved IMF of steers might be closely associated with a reduction in lipolysis hormone signaling due to castration, which might be explained by castration diminishing the negative feedback of the hypothalamic-pituitary-testicular axis [57]. Signal hormones, such as GnRH, estrogen and oxytocin, could affect mitogen-activated protein kinases (MAPK) signaling pathway, which is closely related to the process of adipogenesis [58]. *Mitogen-activated protein kinases 1* (*MAPK1*) expression is significantly decreased, whereas *PPARG* expression increased in the backfat of castrated male pigs compared with IM pigs, implying that MAPK signaling pathway is a vital pathway in fat deposition induced by castration [13]. We found that several genes involved in MAPK signaling pathway, such as *MAP3K1*, an *MAPK kinase 4* (*MKK4*) activator, *RAC2*, a *MAP3K1* activator, *and JUN*, a *c-Jun NH 2-terminal kinase* (*JNK*) activator, were all downregulated in the IMF of steers [59,60]. Interestingly, the JNK/MAPK signaling pathway appears to represses adiposity in mesenchymal stem cells [61]. These findings indicate that the possible molecular mechanism of improving IMF accumulation in steers might be partly attributed to a negative regulatory mechanism of the MAPK signaling pathway.

Hormonal regulation of adipocyte metabolism is a complex process, and miRNA-target regulation is an essential response. A number of miRNAs related to lipid metabolism and adipogenesis were differentially expressed between bulls and steers, which included downregulated miRNAs, such as *miR-224*, *miR-33a*, *miR-1*, and *miR-141*, and upregulated miRNAs, such as *miR-10b*, *miR-193*, and *miR-181b*. *MiR-224* seem to inhibit adipocyte differentiation during early adipogenesis by decreasing *early growth response 2* (*EGR2*) and could regulate fatty acid metabolism by targeting *acyl-CoA synthetase long-chain family member 4* (*ACSL4*) at terminal differentiation [62]. Overexpression of *miR-33a* and *miR-33b* also reduces fatty acid oxidation and insulin signaling in hepatic cell lines by reducing *carnitine O-octaniltransferase* (*CROT*), *CPT1A*,*hydroxyacyl-CoA-dehydrogenase* (*HADHB*), *AMP kinase subunit-α* (*AMPKα*) and *insulin receptor substrate 2* (*IRS2*), which promote fatty acid oxidation and insulin signaling [63]. Upregulation of *miR-1* also occurs during the development of obesity in mice [64], whereas blocking *miR-193b* inhibits brown adipocyte adipogenesis by significantly inducing the expression of myogenic markers [65]. Moreover, *miR-140* markedly increases morbidly obese patients [66], whereas *miR-10b* regulates steatosis by directly targeting *peroxisome proliferator-activated receptor alpha* (*PPARA*), which is important in the cellular metabolic response to fasting [67]. Overexpression of miR-181a also accelerates the accumulation of lipid droplets in pigs by inhibiting the expression of *tumor necrosis factor alpha* (*TNFA*). Thus, our screening of functional miRNAs related to the regulation of IMF deposition after castration is reliable [68].

By negative correlation analysis of the DE miRNA and mRNA profiles with a relatively strict threshold (|log2(FC)|≥1, FDR<0.05; Energy≤-20, Score≥150), a miRNAs targets network was constructed (Fig 7A and 7B). The effects of castration on miRNA and mRNA expression do not appear to have been investigated in IMF tissue in a manner of integrative analysis. In the network, the most important genes targeted by the downregulated miRNAs include *ELOVL5*, *LPL*, *LDLR* and *kinesin superfamily motor protein 17* (*KIF17*), all known to be involved in lipid metabolism and adipogenesis, whereas other important genes targeted by upregulated miRNAs included *HBEGF*, *MAP3K1*, *paired box gene 8* (PAX8), *PAK1*, *RAC2*, *JUN* and *activating transcription factor 3* (ATF3), all known to be involved in hormone signaling pathway and the MAPK signaling pathway. Interestingly, our data on targets validation provided strong evidence that *MAP3K1* and *HBEGF* are targets of *bta-let-7i*, whereas *PAK1* and *RAC2* are targets of *bta-miR-2305* (Fig 7C, 7D, 7E, 7F and 7G). We therefore hypothesize that these miRNAs can target sequences in these genes to regulate the deposition of IMF tissue following castration.

IPA molecular network analysis has also elucidated that there are 9 DE miRNAs and 42 DE mRNAs involved in differentiation of adipocytes and lipid metabolism (Fig 8). More particularly, our data indicate that the DE genes *HBEGF*, *MAP3K1* and DE miRNA *let-7a-5p* have previously been associated with adipogenesis and lipid metabolism (*e*.*g*. MAPK signaling pathway). Among them, *let-7a-5p* and *bta-let -7i* belong to the same miRNA family with seed-pairing regions (GAGGUAG). MiR-143 promotes adipocyte differentiation by targeting *MAPK1* in both human pre-adipocytes and mouse 3T3-L1 cells, indicating that these miRNAs could affect adipogenesis by regulating the *MAPK* signaling pathway [69]. *HBEGF*, one of the genes involved in the GnRH hormone signaling pathway, stimulates the growth of a variety of cells in an autocrine or paracrine manner, and has a close relationship with MAPK in non-transformed human mammary epithelial cells [70]. These findings implying that *bta-let-7i* might affect adipogenesis or lipid metabolism by affecting MAPK signaling pathway, which is supported by our findings from the functional identification of *bta-let-7i* on bovine pre-adipocytes. However, further research is needed to determine whether the *bta-let-7i* promotes bovine pre-adipocytes differentiation by directly targeting *MAP3K1* and *HBEGF*. These results provide evidence for using our methods of identifying the significant genes related to this biological function.

IPA analysis also showed that genes *JUN*, *MAP3K1*, *MAPK1*, *cyclic adenosine monophosphate* (cAMP) *response element-binding protein 1* (*CREB1*) and *PPARA* are probably the most critical ones in the bovine IMF tissues. Two well-characterized signaling pathways, including PPAR signaling pathway and MAPK/ cAMP response element binding protein (CREB) signaling pathway, stood out in our gene interaction hierarchy. PPARA is a nuclear receptor that regulates liver and skeletal muscle lipid metabolism and glucose homeostasis [71]. As a nuclear transcriptional factor, CREB is crucially involved in cell growth and differentiation, and is a phosphorylation substrate of several protein kinase pathways, such as adenylyl cyclase (AC)–cyclic adenosine monophosphate (cAMP)–Camp-dependent protein (PKA), Ca2+–calmodulin-dependent protein kinase (CAMK), MAPK–CREB and phosphatidylinositol-3-kinase (PI3K)–serine/threonine kinase (AKT) [72]. CREB is activated in 3T3-L1 cells treated with conventional differentiation-inducing agents that induce adipogenesis by binding to putative cAMP response elements (CREs) in several adipocyte-specific gene promoters, such as *phosphoenolpyruvate carboxykinase* (*PEPCK*), *fatty acid binding protein* (*FABP*), and *FASN*, and directly modulate their transcription [73].

Our findings indicate that *5-hydroxytryptamine receptor 2A* (*HTR2A*), the top DE gene in the molecular network, was upregulated with at least a 10.28-FC (FDR<0.05) in steers compared with bulls, which functioned in pathway Gap junction. In 3T3-L1 pre-adipocyte cells, tryptophan hydroxylase-1, a rate-limiting enzyme for the synthesis of serotonin (5-HT), is required for their differentiation, and the HTR2A antagonist inhibited adipocyte differentiation [74], which suggests that castration may activate the HTR2A-mediated pathway and consequently improve pre-adipocyte differentiation in the steers. Accordingly, it might be intriguing to experiment on the functionality of these genes in cattle to improve our understanding of the mechanisms causing the varied IMF performance.

## Conclusions

Through transcriptional analysis of the genes and miRNAs expressed in the IMF tissues of steers and bulls in Chinese Qinchuan cattle, we identified the genes, miRNAs and pathways that may affect the IMF phenotype of cattle. These DE genes and miRNAs were functionally related to lipid metabolism, TG biosynthesis, fatty acid metabolism, immune response and hormone signal transduction pathways, suggesting important roles of signaling pathways contributing to improved IMF deposition following castration of bulls. The findings highlight the essential functions of miRNA-target networks in determining IMF accumulation in steers. Combining molecular network and DE genes and miRNAs expression analysis, a molecular network that includes 9 DE miRNAs and 42 DE genes involved in adipocyte differentiation and metabolism of adipocyte tissues has been constructed in the IPA system. Our findings will expand the scope of further studies with genes and miRNAs relevant to bovine IMF deposition. The data provides more insights into the mechanisms to enhance the IMF deposition and meat quality of beef.

## Supporting information

S1 Table Primers used for qRT-PCR validation. (XLS)

S2 Table Statistics of the raw and mapped reads from the RNA-seq analysis of IMF tissue from steers and bulls, respectively. (XLS)

S3 Table List of differentially expressed genes between steers and bulls. (XLS)

S4 Table List of highly enriched GO terms related to the upregulated genes in steers vs. bulls. (XLS)

S5 Table List of highly enriched GO terms related to the downregulated genes in steers vs. bulls. (XLS)

S6 Table List of highly enriched pathways related to the upregulated genes in steers vs. bulls. (XLS)

S7 Table List of highly enriched pathways related to the downregulated genes in steers vs. bulls. (XLS)

S8 Table Summary of the sequencing read alignments to the reference genome. (XLS)

S9 Table Known bovine miRNAs expressed in the IMF from the steers and bulls cattle. (XLS)

S10 Table Candidate novel bovine miRNAs expressed in the IMF from the steers and bulls cattle. (XLS)

S11 Table A total of 101 DE miRNAs and their 534 DE target genes showing negative correlations in steers vs. bulls (Energy ≤-20, Score ≥150). (XLS)

S12 Table Detail information on the molecular networks functionally associated with adipocyte differentiation and adipose tissues metabolism identified by IPA. (XLSX)
